# Topological Properties of Large-Scale Cortical Networks Based on Multiple Morphological Features in Amnestic Mild Cognitive Impairment

**DOI:** 10.1155/2016/3462309

**Published:** 2016-02-23

**Authors:** Qiongling Li, Xinwei Li, Xuetong Wang, Yuxia Li, Kuncheng Li, Yang Yu, Changhao Yin, Shuyu Li, Ying Han

**Affiliations:** ^1^Key Laboratory for Biomechanics and Mechanobiology of Ministry of Education, School of Biological Science & Medical Engineering, Beihang University, Beijing 100191, China; ^2^Center of Alzheimer's Disease, Beijing Institute for Brain Disorders, Beijing 100053, China; ^3^Department of Neurology, Xuanwu Hospital, Capital Medical University, Beijing 100053, China; ^4^Department of Neurology, Tangshan Gongren Hospital, Tangshan 063000, China; ^5^Department of Radiology, Xuanwu Hospital, Capital Medical University, Beijing 100053, China; ^6^Department of Neurology, Hongqi Hospital, Mudanjiang Medical University, Mudanjiang 157011, China

## Abstract

Previous studies have demonstrated that amnestic mild cognitive impairment (aMCI) has disrupted properties of large-scale cortical networks based on cortical thickness and gray matter volume. However, it is largely unknown whether the topological properties of cortical networks based on geometric measures (i.e., sulcal depth, curvature, and metric distortion) change in aMCI patients compared with normal controls because these geometric features of cerebral cortex may be related to its intrinsic connectivity. Here, we compare properties in cortical networks constructed by six different morphological features in 36 aMCI participants and 36 normal controls. Six cortical features (3 volumetric and 3 geometric features) were extracted for each participant, and brain abnormities in aMCI were identified by cortical network based on graph theory method. All the cortical networks showed small-world properties. Regions showing significant differences mainly located in the medial temporal lobe and supramarginal and right inferior parietal lobe. In addition, we also found that the cortical networks constructed by cortical thickness and sulcal depth showed significant differences between the two groups. Our results indicated that geometric measure (i.e., sulcal depth) can be used to construct network to discriminate individuals with aMCI from controls besides volumetric measures.

## 1. Introduction

Mild cognitive impairment (MCI) is considered to be a transitional period between normal aging and Alzheimer's disease (AD), which is a progressive, neurodegenerative disease characterized by cognitive decline greater than expected for one's age and educational level yet not fulfilling the criteria of AD [1]. Amnestic MCI (aMCI), as the most common subtype of MCI, is characterized by primary memory impairments with single or multiple cognitive domains impaired and likely progresses to AD [2–4]. Current studies of aMCI have shown disrupted functional integration [5] and abnormal structural connections between regions [6]. Morphological features have been widely used to characterize brain structures [7, 8] and also served as structural measures to investigate topological properties in large-scale cortical networks [9–11]. Previous studies on large-scale cortical network in MCI mostly used cortical thickness and gray matter volume as descriptors to construct structural network of the human cortex [12, 13].

However, different morphological features reveal different intrinsic properties of cerebral cortex. For example, volumetric measures (i.e., cortical thickness, gray matter volume) reflect the size, density, and arrangement of cells (neurons, neuroglia, and nerve fibers) [14, 15], and surface area is linked to the number of mini columns in the cortical layer [16]. Using large-scale cortical network analysis based on cortical thickness, several studies have found disrupted small-world properties (i.e., lower clustering coefficient and shorter path length) in MCI patients compared to normal controls [13, 17, 18]. A cortical network study using surface area can reveal topological properties of the networks resulting from the concurrent changes between different anatomical regions [10]. In addition, geometric measures (i.e., sulcal depth, curvature, and metric distortion) mainly reflect cortical folding pattern [19–21]. For instance, sulcal depth and curvature measure specific aspects of the cortical geometry, and metric distortion is a wider measure of the overall degree of cortical folding [22]. These geometric measures related to cortical folding may vary with the changes of intrinsic as well as extrinsic connectivity according to the tension theory of the cerebral cortex morphogenesis [19] and could be more suitable descriptors for finding the anatomical-axonal and morphological connectivity correlation [10]. Thus, we assume that geometric measures can be used to construct cortical network that may detect the alterations from structural disconnection in aMCI and show different topological properties compared with volumetric measures (i.e., cortical thickness, gray matter volume, and surface area).

Here, we investigated topological properties of large-scale human cortical network based on graph theory analysis method by employing multiple morphological features in aMCI patients. Then we compared the topological properties of different cortical networks constructed by different morphological features. We expected that topological properties of cortical networks based on geometric measures in aMCI patients may be different from normal controls and can be used to discriminate individuals with aMCI from controls.

## 2. Materials and Methods

### 2.1. Participants

Seventy-two right-handed participants, including thirty-six aMCI and demography matched healthy normal controls, participated in this study. The aMCI participants were recruited from a clinical research program at Xuanwu Hospital, Beijing, China. The healthy normal controls were recruited from the local community through advertisements. This study was approved by the Research Ethics Review Board of Xuanwu Hospital, and written informed consent was obtained from each participant.

All the aMCI participants were identified according to the criteria for amnestic MCI [23–26], which included (a) memory complaint, preferably confirmed by an informant; (b) objective memory impairment, adjusted for age and education; (c) normal or near-normal performance on general cognitive functioning and no or minimum impairment of daily life activities; (d) the Clinical Dementia Rating (CDR) score of 0.5; and (e) not meeting the criteria for dementia according to the DSM-IV (Diagnostic and Statistical Manual of Mental Disorders, 4th Edition, revised). Participants with aMCI were diagnosed by experienced neurologists. Participants were excluded if they met the following clinical characteristics: (a) a clear history of stroke; (b) severe depression that led to mild cognitive impairment (Hamilton Depression Rating Scale score >24 points); (c) other nervous system diseases, which can cause cognitive impairment (such as brain tumors, Parkinson's disease, encephalitis, and epilepsy); (d) cognitive impairment caused by traumatic brain injury; (e) other systemic diseases, which can cause cognitive impairment, such as thyroid dysfunction, severe anemia, syphilis, and HIV; and (f) a history of psychosis or congenital mental growth retardation. Clinical and demographic data for the participants are shown in Table 1.

### 2.2. MRI Data Acquisition

MRI data acquisition was performed on a 3.0 T Siemens scanner by employing a sagittal magnetization-prepared rapid gradient echo (MP-RAGE) sequence with the following imaging parameters: repetition time (*T*_R_) = 1900 ms; echo time (*T*_E_) = 2.2 ms; inversion time = 900 ms; flip angle = 90°; field of view (FOV) = 250 mm × 250 mm; matrix = 256 × 256; 176 slices, thickness = 1.0 mm. Brain MR images were inspected by an experienced neuroradiologist, and no gross abnormalities were observed for any subject.

### 2.3. Cortical Reconstruction and Morphological Features Extraction

**Figure 1:**
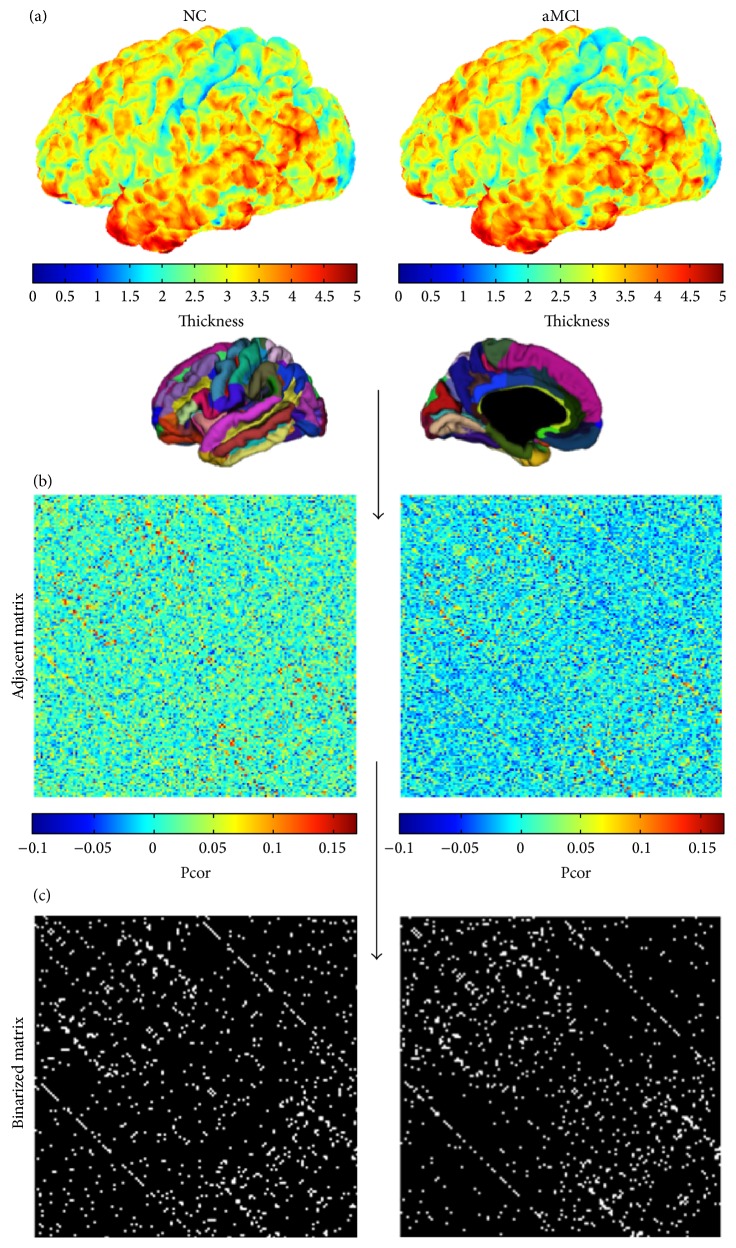
Flowchart for the construction of structural cortical networks. (a) Two representative cortical thickness maps (left for a control subject and right for an aMCI subject) were obtained from anatomical MRI. The color bar indicating the range of thickness is shown on the right. (b) The cortical thickness was mapped into 148 regions and the partial correlation matrices were obtained between regional thicknesses across subjects within each group (left for NC and right for aMCI). The color bar indicating the partial correlation coefficient between regions is shown on the right. (c) The correlation matrices of (c) were thresholded into the binarized matrices (left for NC and right for aMCI) by sparsity of 5%. NC, normal controls.

Both the cortical reconstruction and morphological features extraction were obtained by using the FreeSurfer software (http://surfer.nmr.mgh.harvard.edu/) with a standard cortical automatic handling protocol. First, the data were normalized to a standard anatomical template [27] and corrected for bias-field inhomogeneity. Then the images were skull-stripped using a watershed algorithm [28] and subsequently segmented into subcortical white matter and deep gray matter volumetric structures [29, 30]. The initial tessellation was formed by reconstructing the gray matter/white matter boundary (white surface) and the outer cortical surface (pial surface) [31, 32]. Subsequently, a series of deformable procedures were performed, including surface inflation [31], registration to a spherical atlas [33], and parcellation of the cerebral cortex into units based on gyral and sulcal structures [30]. All reconstructed surfaces were visually inspected for gross-anatomical topological defects. Finally, a variety of morphological features at each vertex on the pial surface were computed, including volumetric (cortical thickness, surface area, and GM volume) and geometric (sulcal depth, metric distortion, and mean curvature) measures, more details seen in this paper [34]. The thickness maps of both NC and aMCI groups are shown in Figure 1(a).

### 2.4. Cortical Network Construction

We employed a cortical scheme comprised of 148 regions from Destrieux Atlas. Cortical networks were built from partial correlation of interregional cortical morphological features. Prior to the correlation analysis, a linear regression was performed at every region to remove the effects of age, gender, and the total morphological feature value for each measure. And the resulting residuals were used to substitute for the raw morphological feature values. In this experimental design, the number of observations (participants, *N* = 36) is smaller than the number of dependent variables (regions, *P* = 148). “Small *N*, large *P*” lead to inaccurate estimations of the covariance matrix [35]. A method based on the Ledoit-Wolf lemma was used to shrink the covariance estimates [36]. Finally, the partial correlation coefficients were computed with R software (http://www.r-project.org/). The partial correlation matrixes (adjacent matrix) of cortical networks constructed by thickness are shown in Figure 1(b).

The adjacent matrix was then binarized to an undirected and unweighted graph as shown in Figure 1(c) (at the sparsity of 5%) using a wide range of sparsity values (from 5% to 35%, step = 0.01). Sparsity of 5% meant that only the strongest 5% of the connections remained and 95% of the connectivity matrices were removed. If the sparsity was less than 5%, the small-world properties were not estimable. And if the sparsity was greater than 35%, more noise would be included in the graph and it would be more like random network [37, 38]. The same sparsity range was applied for all network analyses.

### 2.5. Graph Theoretical Characterization

Graph theory is usually considered an attractive model for the mathematical treatment of cortical network connectivity [39]. In general, a complex network can be represented as a graph *G*, which consists of a set of nodes and a set of edges. Several important parameters of the graph *G* for the connectivity matrices were estimated in this study.

Degree is the number of links connected to the node. Degree of a node “*i*” is defined as

(1) $$\begin{array}{c} k_{i}=\underset{j\in N}{\sum }a_{ij}, \end{array}$$

where *N* is the set of all nodes in the network; *a*_*ij*_ is the connection status between nodes “*i*” and “*j*” and *a*_*ij*_ = 1 when link exists; otherwise *a*_*ij*_ = 0.

The clustering coefficient *C*_*i*_ of a node “*i*” with degree *k*_*i*_ is defined as the ratio of the existing connections (*e*_*i*_) between the node's neighbors and the maximum possible connections between neighbors of the node. The clustering coefficient of node “*i*” is given as

(2) $$\begin{array}{c} C_{i}=\frac{2e_{i}}{k_{i}\left(k_{i}-1\right)}. \end{array}$$

The clustering coefficient is an index of local structure, while the clustering coefficient of the whole network is the average *C*_*i*_ over all nodes

(3) $$\begin{array}{c} C=\frac{1}{N}\overset{N}{\underset{i=1}{\sum }}C_{i}. \end{array}$$

The shortest path length *L*_*i*,*j*_ between two nodes “*i*” and “*j*” of the graph *G* is the smallest number of edges that is required to connect “*i*” and “*j*.” The shortest path length of a node “*i*” can be calculated as the distance between a node “*i*” and all other nodes [37]:

(4) $$\begin{array}{c} L_{i}=\frac{1}{N}\overset{N}{\underset{j=1,\;j\neq i}{\sum }}L_{i,j}. \end{array}$$

The characteristic path length is defined as the mean of path length *L*_*i*,*j*_ over all pairs of nodes:

(5) $$\begin{array}{c} L=\frac{1}{N}\overset{N}{\underset{i=1}{\sum }}L_{i}. \end{array}$$

The small-worldness network parameter *σ* is defined as those with small path length, like random network, and high clustering coefficient networks, much higher than random network. Small-world properties of a given network may be influenced by its intrinsic features, such as the number of nodes, edges, and the degree distribution. Thus, 1000 random networks were generated by using a random rewiring process [40], which preserves the number of nodes, mean degree, and degree distribution. This results in a normalized clustering coefficient *γ* = *C*_*p*_/*C*_rand_ ≫ 1 and a normalized path length *λ* = *L*_*p*_/*L*_rand_ ≈ 1. Then a simple quantitative measurement of small-worldness *σ* is acquired [41]:

(6) $$\begin{array}{c} \sigma =\frac{\gamma }{\lambda }. \end{array}$$

The real cortical network *G* is considered to be a small-world network if it meets the following criteria [37]:

(7) $$\begin{array}{c} \sigma =\frac{\gamma }{\lambda }>1. \end{array}$$

Betweenness centrality is a measure of network hubs that are crucial to efficient communication. BC is defined as the ratio of the number of shortest path passing through node “*i*” to the total number of shortest paths between pairs of nodes “*j*” and “*k*”:

(8) BCi=∑j, k∈Nj≠kρj,kiρj,k,

where *ρ*_*j*,*k*_ is the number of shortest paths between “*j*” and “*k*” and *ρ*_*j*,*k*(*i*)_ is the number of shortest path between “*j*” and “*k*” that passes through “*i*.” For further comparison, the betweenness BC_*i*_ would be normalized as bc_*i*_ = BC_*i*_/BC, where BC is the average betweenness of the network. Cortex regions were defined as hubs, whose betweenness values were more than twice the average betweenness of the network (bc_*i*_ > 2).

### 2.6. Statistical Analysis

Two-sample *t*-test was used to test the demographics, in which gender was converted into a virtual variable. To test the statistical significance of the between-group differences in the parameters of the cortical networks, a nonparametric permutation test was employed [42]. In this permutation test, we calculated possible values of the test statistic on a reference distribution after repeatedly rearranging the observed data from NC and aMCI groups. First, characteristics of the cortical network, such as *C*_*p*_, *L*_*p*_, and bc_*i*_, were calculated for NC and aMCI groups, respectively. Then NC and aMCI data were mixed. From the mixed data, the same number of subjects as aMCI patients was randomly chosen to be considered as aMCIs and the rest to be NCs. Next, partial correlation matrix for each randomized group was recalculated and corresponding binarized matrix was obtained using the same sparsity as in the real cortical networks. Third, network parameters for each randomized group were computed. This process was repeated 1000 times and the 95 percentile points of each distribution used as the critical values for a one-tailed procedure were repeated at every sparsity value of the cortical networks.

## 3. Results

### 3.1. Demographics

Two-sample *t*-test was used to test the demographics, in which gender was converted into a virtual variable, and results are shown in Table 1. There were no significant differences in gender, age, or years of education between aMCI and NC. Groups for aMCI and NC showed significant differences in MMSE and MoCA scores (*p* < 0.01).

### 3.2. Small-World Properties of Cortical Networks

**Figure 2:**
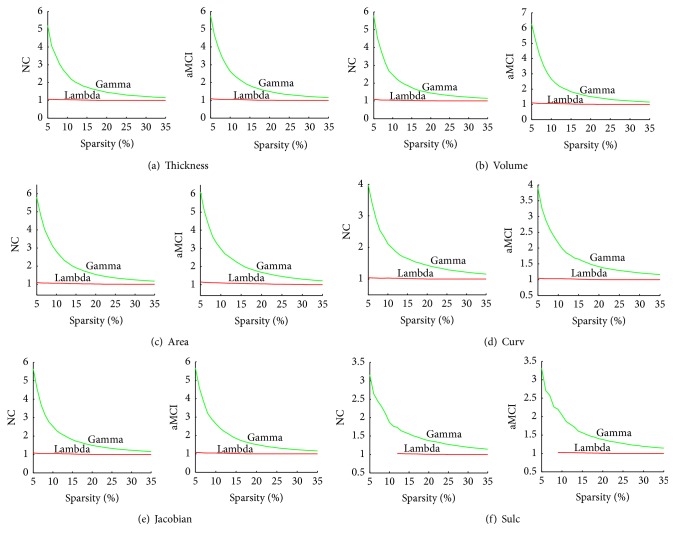
Small-world properties of volumetric measures networks and geometric measures networks. The graph shows the normalized characteristic path length (lambda, *λ* = *L*_*p*_/*L*_rand_) and clustering coefficients (gamma, *γ* = *C*_*p*_/*C*_rand_ ≫ 1) over a range of sparsity values (5% ≤ sparsity ≤ 35%). All the networks have *γ* ≫ 1 (green lines) and *λ* ≈ 1 (red lines), which imply small-world properties. (a) The values of gamma and lambda in NC and aMCI of cortical thickness networks. (b) The values of gamma and lambda in NC and aMCI of GM volume networks. (c) The values of gamma and lambda in NC and aMCI of surface area networks. (d) The values of gamma and lambda in NC and aMCI of mean curvature networks. (e) The values of gamma and lambda in NC and aMCI of metric distortion (Jacobian) networks. (f) The values of gamma and lambda in NC and aMCI of sulcal depth networks. Thickness, cortical thickness. Volume, gray matter volume. Area, surface area. Curv, mean curvature. Sulc, sulcal depth. NC, normal controls.

Compared with random networks, small-world networks had higher clustering coefficients and similar characteristic path length. Over a range of sparsity values (5% ≤ sparsity ≤ 35%), clustering coefficient and characteristic path length were calculated for both the NC and aMCI networks based on different morphological features. The small-world attributes of the networks are shown in Figure 2. Compared with matched random networks which had the same number of nodes and degree distribution, all morphological networks had similarly characteristic path length (*λ* ≈ 1) and larger clustering coefficients (*γ* ≫ 1) in both NC and aMCI networks. Compared with NC, aMCI showed slightly larger small-world characteristics (larger *σ*) in the cortical networks obtained for volumetric measures (cortical thickness and GM volume) and there were no great differences between NC and aMCI cortical networks based on surface area and geometric measures (mean curvature, metric distortion, and sulcal depth).

### 3.3. Abnormal Changes in Nodal Betweenness Centrality

As crucial components required for efficient communication in a network, hubs regulated information flow and played a key role in network resilience against attacks. To study the nodal characteristics, the cortical networks were constructed at certain sparsity of 11%. This sparsity ensured that all regions were included in the cortical networks while minimizing the number of false-positive paths. Based on the results, some regions were identified as hubs in the cortical network of both the NC and aMCI groups. Details of the hub regions in the cortical networks are shown in Table 2.

**Figure 3:**
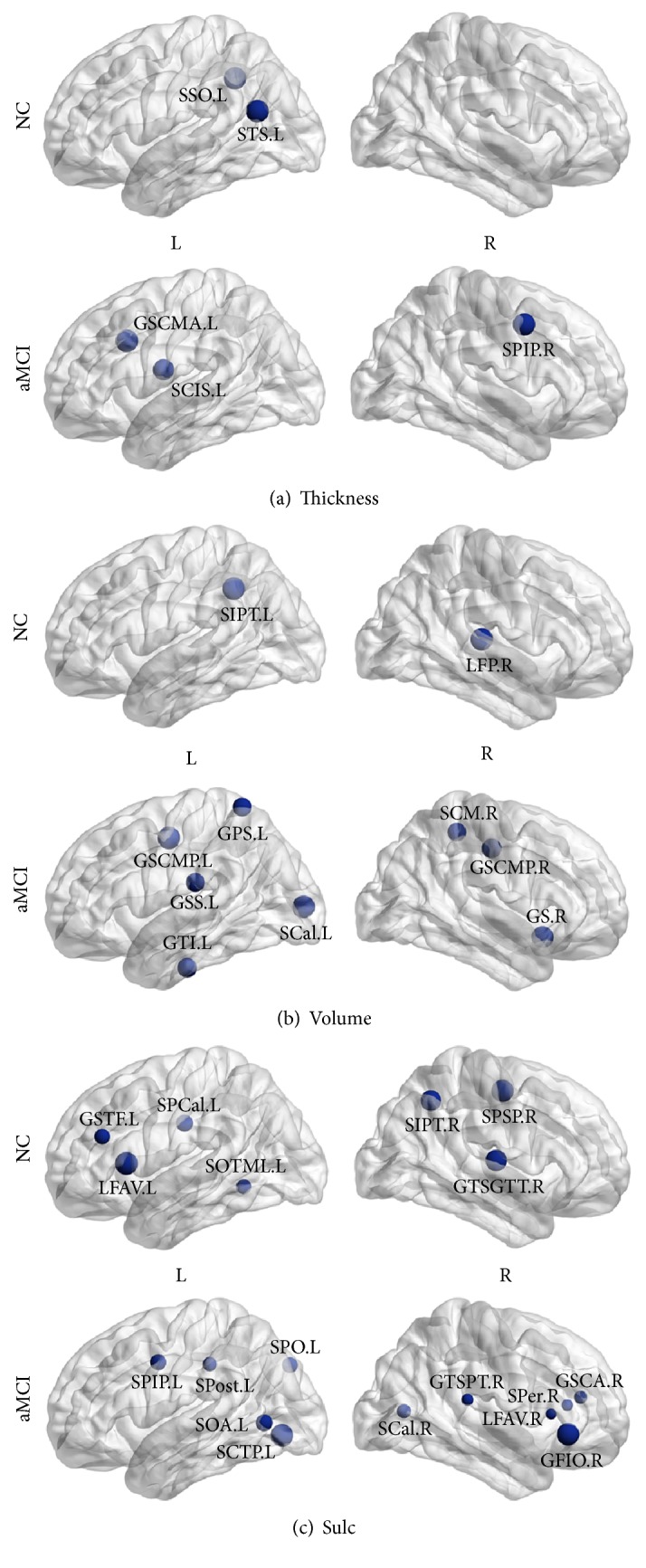
Hubs regions in cortical networks. Global hub regions derived from normalized nodal betweenness centrality in NC and aMCI. The blue spheres indicate the global hubs whose betweenness is more than twice the average betweenness of the network. (a) Global hubs in cortical thickness networks. (b) Global hubs in gray matter volume networks. (c) Global hubs in sulcal depth networks. Thickness, cortical thickness. Volume, gray matter volume. NC, normal controls. For the abbreviations of regions, see Table 2.

In this study, the identified hub in networks based on volumetric measures, as shown in Figures 3(a) and 3(b), was involved in the frontal, temporal, parietal, and insula association cortex in the NC and temporal lobe, superior parietal lobule, cingulate cortex, precentral sulcus, callosum, and insula in the aMCI. High betweenness in network based on geometric measures was similar to volumetric measures. It was worth noting that hubs in networks using sulcal depth as descriptor included frontal polar, lingual sulcus, medial occipitotemporal sulcus, precentral sulcus, temporal gyrus (Heschl), and corpus callosum in NC group. And in aMCI group, regions included collateral sulcus, precentral sulcus, postcentral sulcus, temporal-occipital incisures, frontal gyrus, and corpus callosum (Figure 3(c)).

**Figure 4:**
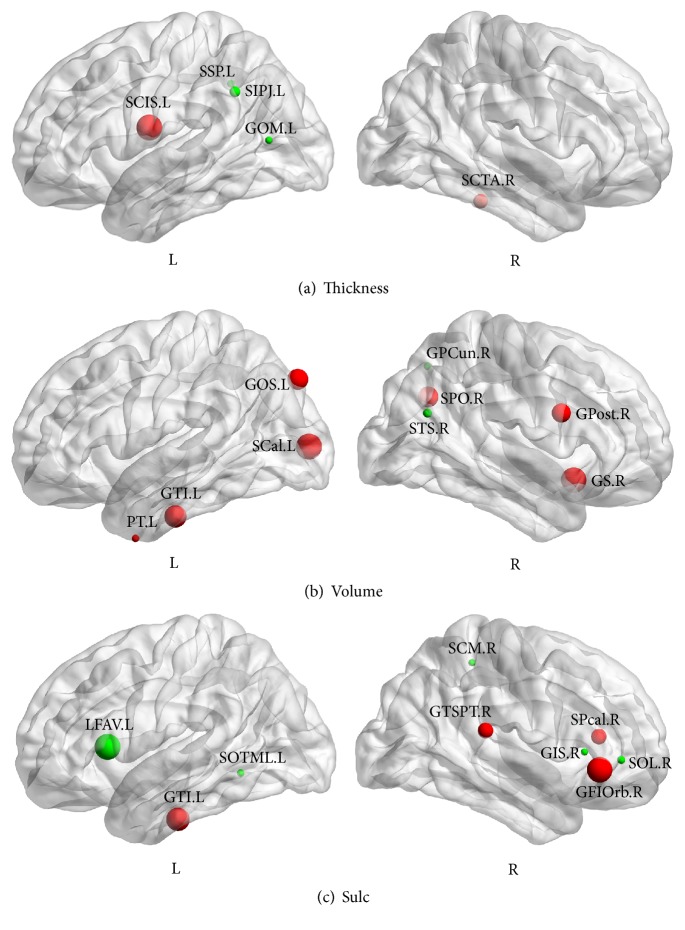
Abnormal changes in nodal betweenness centrality. The graph shows significant difference (*p* < 0.05) in betweenness between two groups. The green spheres indicate significant decreases in between-group nodal centrality. The red spheres indicate significant increases in between-group nodal centrality. (a) Abnormal changes in cortical thickness networks. (b) Abnormal changes in gray matter volume networks. (c) Abnormal changes in sulcal depth networks. Thickness, cortical thickness. Volume, gray matter volume. NC, normal controls. For the abbreviations of regions, see Table 2.

Permutation test was used to detect the significant differences in betweenness between NC and aMCI. Regions showing significant increase (*p* < 0.05) in the betweenness of cortical networks using volumetric measures in aMCI patients included collateral sulcus, occipital gyrus, temporal gyrus, temporal pole, parietooccipital sulcus, postcentral gyrus, and subcallosal gyrus. And decreased betweenness (*p* < 0.05) regions were located in subparietal sulcus, middle occipital gyrus, precuneus, and superior temporal sulcus as shown in Figures 4(a) and 4(b). Betweenness in inferior temporal gyrus, superior temporal gyrus, inferior frontal gyrus, and pericallosal sulcus showed significant increase (*p* < 0.05) in network constructed by sulcal depth in aMCI patients. And betweenness in lateral sulcus, medial occipitotemporal sulcus, lateral occipitotemporal sulcus, cingulate sulcus, and short insular gyri significantly decreased (*p* < 0.05).

### 3.4. Comparing Networks from Different Morphological Features between Groups

#### 3.4.1. Volumetric Measures

**Figure 5:**
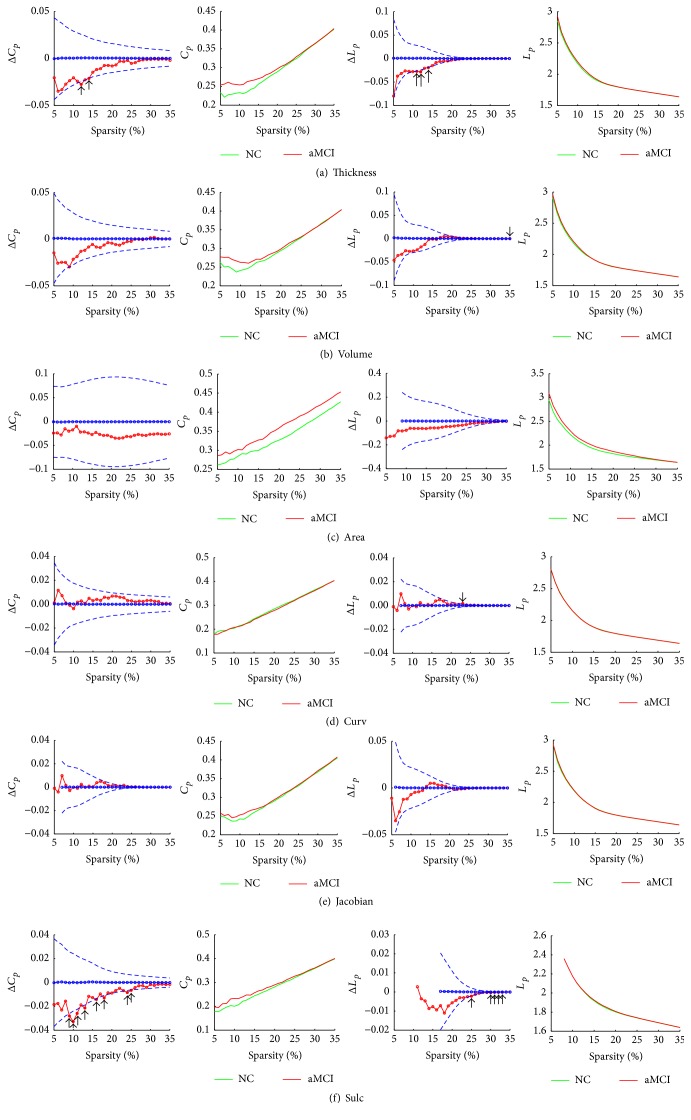
Between-group differences in clustering coefficient (*C*_*p*_) and characteristic path length (*L*_*p*_) of different morphological features based networks. The graph shows the differences in *C*_*p*_ and *L*_*p*_ between NC and aMCI as a function of sparsity of geometric measures networks. The blue lines represent the mean values (open circles) and 95% confidence intervals of the between-group differences obtained from 1000 permutation tests at each sparsity value. The arrows indicate significant (*p* < 0.05) difference in *C*_*p*_ or *L*_*p*_ between the two groups. (a) Between-group differences in *C*_*p*_ and *L*_*p*_ as a function of sparsity of cortical thickness networks. (b) Between-group differences in *C*_*p*_ and *L*_*p*_ as a function of sparsity of gray matter volume networks. (c) Between-group differences in *C*_*p*_ and *L*_*p*_ as a function of sparsity of surface area networks. (d) Between-group differences in *C*_*p*_ and *L*_*p*_ as a function of sparsity of mean curvature networks. (e) Between-group differences in *C*_*p*_ and *L*_*p*_ as a function of sparsity of metric distortion (Jacobian) networks. (f) Between-group differences in *C*_*p*_ and *L*_*p*_ as a function of sparsity of sulcal depth networks. Thickness, cortical thickness. Volume, gray matter volume. Area, surface area. Curv, mean curvature. Sulc, sulcal depth. NC, normal controls.

As shown in Figures 5(a) and 5(b), clustering coefficient and characteristic path length were higher in the structural cortical networks obtained from volumetric measures (both cortical thickness and GM volume) of aMCI. A permutation test was used to detect the between-group differences. The arrows indicated the significant differences between NC and aMCI in the clustering coefficient (*p* < 0.05) of networks constructed by cortical thickness at the sparsity of 12% and 14% as shown in Figure 5(a). Significant differences in characteristic path length (*p* < 0.05) of networks constructed by cortical thickness had been detected between NC and aMCI at the sparsity of 11%, 12%, and 14%. In the cortical networks obtained from GM volume, as shown in Figure 5(b), no significant differences were found in clustering coefficient between NC and aMCI (*p* > 0.05). Only at the sparsity of 35% was a significant difference found in characteristic path length (*p* < 0.05). Our findings provided further evidence for which networks constructed by cortical thickness had a small-world characteristic loss in aMCI.

In Figure 5(c), the clustering coefficient and characteristic path length were much larger for aMCI in cortical network using surface area as descriptor. However, no significant differences (*p* > 0.05) were found in all permutation tests for small-world properties of cortical network based on surface area.

#### 3.4.2. Geometric Measures

Small-world properties of cortical network using sulcal depth were very similar to properties in network using thickness for both NC and aMCI. As shown in Figure 5(f), the clustering coefficient was higher for aMCI, and the characteristic path length had no much difference between aMCI and NC. Statistical analysis further revealed significant differences in the clustering coefficient (*p* < 0.05) at 9% ≤ sparsity ≤ 11%, sparsity = 13%, 16%, and 18%, and sparsity = 24% and 25%. Significant differences were found in the characteristic path length between NC and aMCI at the range of sparsity values (sparsity = 25% and 30% ≤ sparsity ≤ 33%).

In Figure 5(e), small-world properties analysis using metric distortion as a descriptor showed similar results to properties in network based on cortical thickness. As shown in Figure 5(e), the clustering coefficient was larger for the aMCI compared with NC subjects. What is more, the characteristic path length had no much difference between NC and aMCI. However, statistical analysis revealed no significant differences (*p* > 0.05) in all the topological parameters over the whole range of sparsity values. Similar to metric distortion, no significant differences were found when using mean curvature as descriptor in cortical network (*p* > 0.05).

## 4. Discussion

In this study, we explored the properties of large-scale human brain cortical networks using multiple morphological features (including 3 volumetric measures, cortical thickness, surface area, and gray matter volume, and 3 geometric measures, sulcal depth, metric distortion, and mean curvature) based on graph theory analysis in cognitively normal older adults and amnestic mild cognitive impairment (aMCI) patients. We found that all networks constructed by these morphological features showed small-world properties which implied high efficiency of information transformation in human cognition. Properties in networks constructed by cortical thickness and sulcal depth showed significant differences between NC and aMCI patients. Besides, regions showing significant differences mainly located in the medial temporal lobe and supramarginal and right inferior parietal lobe. Our results indicated that geometric measure (i.e., sulcal depth) can be used to construct network to discriminate individuals with aMCI from controls besides volumetric measures and provided new insights into the study of the pathophysiological mechanism of amnestic MCI.

Previous studies have demonstrated that the cortical thickness and GM volume can be used as morphological descriptors to study the complex cortical networks, and networks based on the cortical thickness and GM volume followed the small-world properties [10–12, 43, 44]. Similar to previous studies, networks based on volumetric measures showed altered small-world properties (i.e., increased clustering coefficient and path length) in aMCI patients compared with NC subjects. Short path length and high clustering coefficient in cortical network mean effective and rapid transfers of information between and across remote regions that are believed to constitute the basis of cognitive processes. Large *σ* means an optimal balance between local specialization and global integration. The cortical thickness changes are related to myelination of gray matter or the underlying white matter, as we know damage of myelin sheath is often associated with decreased functional efficiency. Here, we found longer path length and higher clustering coefficient in aMCI that may indicate a disturbance of the normal balance [45].

Consistent with the volumetric measures, all networks based on geometric measures also followed the small-world properties but less optimal small-worldness in aMCI network, while properties in network constructed by sulcal depth showed much more significant differences between NC and aMCI patients compared with properties in networks based on other geometric measures through a range of sparsity values. Previous studies have demonstrated that geometric differences are predominantly linked with the development of neuronal connections and cortical pattern of connectivity [19, 46] and are thus a marker for cerebral development or abnormal cortical connectivity due to disorders. Here networks constructed by sulcal depth in aMCI with less optimal small-worldness implied abnormal structural connections between specific regions in aMCI patients.

Previous studies indicated that hubs were mainly in regions of the parietal, temporal, and frontal heteromodal association cortex (SPL, SMG, MTG, STG, IFG, and SFG) and highly connected primary motor cortex (PrCG) [45]. Hubs in this study were predominately in frontal, temporal, parietal, and insula association cortex in NC of networks based on volumetric measures. Many previous studies ignore the insula when constructing cortical network because the insula is covered by other lobes. Compared with NC, there were more hubs in aMCI involved intemporal lobe, superior parietal lobule, cingulate cortex, precentral sulcus, callosum, and insula. In networks based on sulcal depth, hub regions in NC were compatible with previous studies of functional and structural cortical network [47]. These hub regions, which are considered to be the substrates of human cognition and consciousness, are in the association cortex that receives convergent inputs from multiple other cortical regions [12]. And in networks based on sulcal depth, hub regions in aMCI had more hubs compared with NC, which was similar to regions in networks based on volumetric measures.

Evidences from previous studies have shown the shrunk brain regions in aMCI patients located in parahippocampal gyrus, medial temporal lobe, entorhinal cortex, cingulum, insula, and thalamus [48, 49]. Our results were partially consistent with previous studies. Abnormal changes in the temporal, occipital gyrus and cingulated sulcus in aMCI group have been reported as being related to memory performance. What is more, significantly higher nodal centrality in aMCI was considered as increased functional connectivity occurred in various brain regions [50]. This may serve as a compensatory mechanism that enables patients with aMCI to use other additional resources to maintain normal cognitive performance [51, 52]. The abnormal characteristics of the cortical networks observed in aMCI may reflect anatomical structural abnormalities. Our findings may contribute to an understanding of the cerebral organization in aMCI.

Some limitations should be addressed in the future. Firstly, several studies have demonstrated that network resolution has an effect on topological properties of human neocortex by using volumetric measures as descriptors of anatomical connectivity [10, 53, 54]. In our network analysis, we only used 148 nodes to construct the network. In the future, it is interesting to investigate the relationship between network resolution and topological properties of human neocortex by using geometric measures. Secondly, topological properties of a given network may be influenced by intrinsic features of that network, such as the number of nodes, number of connections, and degree distribution. To counteract these effects, we used random networks with the same number of nodes and edges as surrogates to normalize the corresponding graph measures. Without any correction, the small-world index cannot be used to compare the small-worldness of different empirical networks. However, random surrogates may increase the sensitivity to differences in nodes number and degrees for the commonly used small-world index [55]. The minimum spanning tree (MST) [56], a mathematically defined and unbiased subnetwork, provides similar information about network topology as conventional graph measures. It is noted that the MST discards all loop connections that the clustering coefficient and path length in the small-world index are highly correlated. Several network characteristics such as modularity, hierarchy, and rich club cannot be interpreted with the MST. There is still no optimal method to normalize network measures. Thirdly, different thresholding may lead to different network topological organizations [47]. Notably, connectivity values often vary depending on subjects and conditions, which can result in differences in average degree when using the same threshold for all networks. In the future, it is important to study the optimal thresholding methods in constructing networks.

## 5. Conclusions

This work demonstrated that besides cortical thickness and gray matter volume, sulcal depth can also be used to study the topological properties of cortical networks. We found that networks based on both the volumetric measures and geometric measures showed small-world properties and properties in these networks were different from aMCI to NC. Notably, properties in cortical network constructed by sulcal depth showed significant differences between the two groups. Our results indicate that geometric measure (sulcal depth) can be used to construct network to discriminate individuals with aMCI from controls.

## Figures and Tables

**Table 1:** Subject demographics.

	aMCI (*n* = 36)	Control (*n* = 36)	*p* value
Gender (M/F)	14/22	15/21	0.813
Age	66.0 ± 8.7 (50–83)	63.9 ± 6.1 (56–79)	0.258
Education	10.2 ± 4.4 (2–21)	10.7 ± 3.2 (5–17)	0.651
MMSE	24.4 ± 3.2 (17–30)	28.1 ± 1.7 (20–30)	**<0.001**
MoCA	20.6 ± 3.7 (15–27)	26.4 ± 2.4 (18–30)	**<0.001**

Age, education, MMSE, and MoCA data are expressed as mean ± SD (range). No significant differences were between two groups in gender, age, and education years. Groups for aMCI and NC showed significant differences in MMSE and MoCA scores (*p* < 0.01). Statistical *p* value was analyzed using two-sample *t*-test, in which gender was converted into a virtual variable.

**Table 2:** The abbreviations of Destrieux Atlas.

Index	Long name	Abbreviations
1	Frontomarginal gyrus (of Wernicke) and sulcus	GSF
2	Inferior occipital gyrus (O3) and sulcus	GSOI
3	Paracentral lobule and sulcus	GSP
4	Subcentral gyrus (central operculum) and sulci	GSS
5	Transverse frontopolar gyri and sulci	GSTF
6	Anterior part of the cingulate gyrus and sulcus (ACC)	GSCA
7	Middle-anterior part of the cingulate gyrus and sulcus (aMCC)	GSCMA
8	Middle-posterior part of the cingulate gyrus and sulcus (pMCC)	GSCMP
9	Posterior-dorsal part of the cingulate gyrus (dPCC)	GCPD
10	Posterior-ventral part of the cingulate gyrus (vPCC, isthmus of the cingulate gyrus)	GCPV
11	Cuneus (O6)	GC
12	Opercular part of the inferior frontal gyrus	GFIOper
13	Orbital part of the inferior frontal gyrus	GFIOrb
14	Triangular part of the inferior frontal gyrus	GFIT
15	Middle frontal gyrus (F2)	GFM
16	Superior frontal gyrus (F1)	GFS
17	Long insular gyrus and central sulcus of the insula	GILSCI
18	Short insular gyri	GIS
19	Middle occipital gyrus (O2, lateral occipital gyrus)	GOM
20	Superior occipital gyrus (O1)	GOS
21	Lateral occipitotemporal gyrus (fusiform gyrus, O4-T4)	GOTLF
22	Lingual gyrus, lingual part of the medial occipitotemporal gyrus (O5)	GOTML
23	Parahippocampal gyrus, parahippocampal part of the medial occipitotemporal gyrus (T5)	GOTMP
24	Orbital gyri	GO
25	Angular gyrus	GPIA
26	Supramarginal gyrus	GPIS
27	Superior parietal lobule (lateral part of P1)	GPS
28	Postcentral gyrus	GPost
29	Precentral gyrus	GPCen
30	Precuneus (medial part of P1)	GPCun
31	Straight gyrus, gyrus rectus	GR
32	Subcallosal area, subcallosal gyrus	GS
33	Anterior transverse temporal gyrus (of Heschl)	GTSGTT
34	Lateral aspect of the superior temporal gyrus	GTSL
35	Planum polare of the superior temporal gyrus	GTSPP
36	Planum temporale or temporal plane of the superior temporal gyrus	GTSPT
37	Inferior temporal gyrus (T3)	GTI
38	Middle temporal gyrus (T2)	GTM
39	Horizontal ramus of the anterior segment of the lateral sulcus (or fissure)	LFAH
40	Vertical ramus of the anterior segment of the lateral sulcus (or fissure)	LFAV
41	Posterior ramus (or segment) of the lateral sulcus (or fissure)	LFP
42	Occipital pole	PO
43	Temporal pole	PT
44	Calcarine sulcus	SCal
45	Central sulcus (Rolando's fissure)	SCen
46	Marginal branch (or part) of the cingulate sulcus	SCM
47	Anterior segment of the circular sulcus of the insula	SCIA
48	Inferior segment of the circular sulcus of the insula	SCII
49	Superior segment of the circular sulcus of the insula	SCIS
50	Anterior transverse collateral sulcus	SCTA
51	Posterior transverse collateral sulcus	SCTP
52	Inferior frontal sulcus	SFI
53	Middle frontal sulcus	SFM
54	Superior frontal sulcus	SFS
55	Sulcus intermedius primus (of Jensen)	SIPJ
56	Intraparietal sulcus (interparietal sulcus) and transverse parietal sulci	SIPT
57	Middle occipital sulcus and lunatus sulcus	SOML
58	Superior occipital sulcus and transverse occipital sulcus	SOST
59	Anterior occipital sulcus and preoccipital notch (temporooccipital incisure)	SOA
60	Lateral occipitotemporal sulcus	SOTL
61	Medial occipitotemporal sulcus (collateral sulcus) and lingual sulcus	SOTML
62	Lateral orbital sulcus	SOL
63	Medial orbital sulcus (olfactory sulcus)	SOMO
64	Orbital sulci (H-shaped sulci)	SOHS
65	Parietooccipital sulcus (or fissure)	SPO
66	Pericallosal sulcus (S of corpus callosum)	SPer
67	Postcentral sulcus	SPost
68	Inferior part of the precentral sulcus	SPIP
69	Superior part of the precentral sulcus	SPSP
70	Suborbital sulcus (sulcus rostrales, supraorbital sulcus)	SSO
71	Subparietal sulcus	SSP
72	Inferior temporal sulcus	STI
73	Superior temporal sulcus (parallel sulcus)	STS
74	Transverse temporal sulcus	STT
